# Heterogeneity of antibody-secreting cells infiltrating autoimmune tissues

**DOI:** 10.3389/fimmu.2023.1111366

**Published:** 2023-02-21

**Authors:** Diane Giovannini, Aude Belbezier, Athan Baillet, Laurence Bouillet, Mitsuhiro Kawano, Chantal Dumestre-Perard, Giovanna Clavarino, Johan Noble, Jacques-Olivier Pers, Nathalie Sturm, Bertrand Huard

**Affiliations:** ^1^ Department of Pathology, Grenoble University Hospital, Grenoble, France; ^2^ Translational Research in Autoimmunity and Inflammation Group (TRAIG), Translational Innovation in Medicine and Complexity (TIMC), University Grenoble-Alpes, CNRS Unité mixte de recherche (UMR) 5525, Grenoble, France; ^3^ Department of Internal Medicine, Grenoble University Hospital, Grenoble, France; ^4^ Department of Rheumatology, Grenoble University Hospital, Grenoble, France; ^5^ Department of Rheumatology, Kanazawa University Hospital, Kanazawa, Japan; ^6^ Immunology Laboratory, Grenoble University Hospital, Grenoble, France; ^7^ Department of Nephrology, Grenoble University Hospital, Grenoble, France; ^8^ B Lymphocytes, Autoimmunity and Immunotherapies, Brest University, INSERM, UMR1227, Brest, France; ^9^ Odontology Unit, Brest University Hospital, Brest, France

**Keywords:** plasma cells, antibodies, autoimmunity, tissues, treatment

## Abstract

The humoral response is frequently dysfunctioning in autoimmunity with a frequent rise in total serum immunoglobulins, among which are found autoantibodies that may be pathogenic by themselves and/or propagate the inflammatory reaction. The infiltration of autoimmune tissues by antibody-secreting cells (ASCs) constitutes another dysfunction. The known high dependency of ASCs on the microenvironment to survive combined to the high diversity of infiltrated tissues implies that ASCs must adapt. Some tissues even within a single clinical autoimmune entity are devoid of infiltration. The latter means that either the tissue is not permissive or ASCs fail to adapt. The origin of infiltrated ASCs is also variable. Indeed, ASCs may be commonly generated in the secondary lymphoid organ draining the autoimmune tissue, and home at the inflammation site under the guidance of specific chemokines. Alternatively, ASCs may be generated locally, when ectopic germinal centers are formed in the autoimmune tissue. Alloimmune tissues with the example of kidney transplantation will also be discussed own to their high similarity with autoimmune tissues. It should also be noted that antibody production is not the only function of ASCs, since cells with regulatory functions have also been described. This article will review all the phenotypic variations indicative of tissue adaptation described so for at the level of ASC-infiltrating auto/alloimmune tissues. The aim is to potentially define tissue-specific molecular targets in ASCs to improve the specificity of future autoimmune treatments.

## 1 Introduction

In autoimmune diseases, the immunosuppressive treatments based on steroids and/or anti-mitotic agents used for decades are timely replaced by more immune selective drugs. These new therapies may be in the form of antagonist and cytotoxic antibodies. Alternatively, solubilized receptors may also be used for antagonism purposes. Very recently, T cells transduced with chimeric antigen receptors (CAR) originating from the oncology field has also entered the game. Regarding humoral immunity, three drugs targeting distinct molecules but achieving a quite similar immunosuppressive function have been approved for clinical usage. The first approval concerns rheumatoid arthritis in 2006 with a depleting antibody directed against the CD20 molecule, an ubiquitous receptor expressed on mature B cells (1). The initial discovery came from a study testing a cytotoxic chimeric antibody, rituximab, in patients suffering from B-cell lymphoma. The drug was found highly effective, but most important for our present consideration a patient cosuffering from rheumatoid arthritis (RA) also showed benefits at the autoimmune level (2). Hence, a new therapeutic wave in autoimmunity was launched, the B-cell depletion. This approval was extended to granulomatosis polyangiitis and pemphigus vulgaris in 2014 and 2018, respectively (3, 4). Ocrelizumab, the second generation humanized anti-CD20 was also approved in multiple sclerosis in 2017 (5). The second approval concerns systemic lupus erythematosus (SLE) in 2011, and is based on an antagonist antibody, belimumab, directed against the B-cell activation factor from the TNF superfamily (BAFF) (6). BAFF (TNFSF13b) is one of the latest identified members of this superfamily (7). Precisely, BAFF acts extramedullary on developing B cells at their transitional stage to provide a signal enabling them to reach their final mature stage. It does so by stimulating the BAFF receptor (BAFF-R) (8). BAFF-deficient animals gave a striking phenotype with the almost complete disappearance of the mature B-cell pool. Such a result was the proof of concept to design BAFF antagonism in the B-cell depletion era. Another humanized BAFF, tabalumab, is also under development (9). The third approval concerns neuromyelitis optica (NMO) with a depleting antibody, inebilizumab, against the CD19 receptor (10). Other drugs targeting B cells are also tested in autoimmune diseases such as the depleting antibody ianalumab targeting the BAFF-R, the negatively signaling epratuzumab targeting CD22, and atacicept a soluble form of the TACI receptor antagonizing BAFF and the related member from the TNF superfamily a proliferation inducing ligand (APRIL, TNFSF13) (11–13). Note that a second soluble form of TACI, telitacept, is currently under development with a first recent approval in SLE (14). According to the European Union and United States clinical trial registers, 16 different autoimmune diseases are tested or announced to be with at least one of these drugs in 2022. More precisely, 13 diseases are targeted with one anti-CD20, 7 with one anti-BAFF, 4 with the anti-BAFF-R, 3 with the anti-CD19, 1 with the anti-CD22 and 2 with atacicept. The anti-CD38 antibody, daratumumab, coming from the hemato-oncology field and successfully used to treat multiple myeloma (MM), the most common plasma cell (PC)-derived tumor, has also entered the autoimmunity field for refractory patients (15). The proteasome inhibitor bortezomib has been tested in a large spectrum of antibody-mediated autoimmune diseases (16). The study is now completed and results are awaited. Finally, inhibitors of B-cell specific kinases such as the Bruton tyrosine kinase used to treat B-cell malignancies are also under investigations (17).

Antibody-secreting cells (ASCs) may be of two kinds, the plasmablast (PB) representing the first effector stage during the B-cell differentiation process by its capacity to secrete antibodies and the terminally differentiated plasma cell (PC). PB and PC are short- and long-lived, respectively. The different stages and associated phenotypes of B-cell differentiation has been recently reviewed by others (18). A common marker used to characterize ASCs is the high expression of CD38. The coexpression of CD38 and CD138 is further considered to represent the final PC stage, at least in the human system (19). One important concern regarding CD19, CD20, CD22, BAFF-R and their targeting in autoimmunity is their temporal regulation of expression. Indeed, expression of all these receptors is gradually lost upon differentiation of B cells into effector ASCs. Expression is totally lost on PCs, while they may still be present but at a downregulated level on PBs. This implies that these targets will lead to depletion at best of short-lived PBs, and will leave untouched PC already differentiated in patients before diagnosis and treatment. Notably, these treatments will remove all B cells, precursors of ASCs. The COVID 19 taught us that such profound depletion may be unwanted in autoimmune patients, when an urgent vaccination is needed to face emerging infectious agents (20). The similar expression pattern for CD19, CD20 and CD22 is explained by the fact that they are all under the control of the transcription repressor B lymphocyte-induced maturation protein (Blimp-1), a master activator of PC differentiation (21). It should be noted that CD19 may be considered as an outlier in this family, since CD19 has been observed at the surface of ASCs (22). CD19 expression on PCs may concern a subset of cells considered to represent an immature stage of PC (23). Others reported that CD19 may be lost earlier at the PB stage (24). There are currently no data regarding BAFF-R regulation by Blimp1. However, it is clear that BAFF-R undergoes downregulation in germinal center B cells (25–27). It is very much likely that BAFF-R follows the same transcriptional control considering its highly similar expression pattern compared to CD20 and CD22 and its absence of function in PC biology (28). The two other receptors for BAFF and APRIL, TACI and BCMA, have a different expression pattern. They are not expressed on naive mature B cells, but appear after antigen encounter. BCMA is ubiquitous on PCs and key for their survival as demonstrated in genetically deficient mice (29). TACI has been defined by others as enigmatic (30). This is definitely the case when one considers that TACI has two different isoforms harboring different subcellular localizations, the positive or negative signaling ability reported, and an uncommon ligand-independent activation mode (31, 32). TACI expression has been best studied in MM with a gene expression profile obtained with TACI^+^ MM cells consistent with terminally differentiated bone marrow (BM) PCs (33, 34). TACI is expressed on healthy ASCs, at least a subset, and has a survival role for these cells (35). BCMA and TACI expression renders BAFF antagonism potentially efficient to target PCs. However, this has not been observed experimentally (36). One explanation to this is the low affinity reported for BAFF with BCMA (37, 38). On the contrary to BAFF, APRIL is a key PC survival factor (28). Hence, atacicept is so far the only biotherapy that may act on established PCs. In an animal model of antibody-mediated autoimmunity, atacicept was found more active than BAFF-only blockade (39). The anti-CD38 daratumumab may look promising. However, one should be aware that CD38 is not only expressed in immune cells including ASCs. In particular, it is expressed by liver stellate cells and anti-CD38 treatments have been associated to a rise in liver enzymes (40, 41). Another important issue regarding PC targeting is their quiescent state rendering them non susceptible to anti-mitotic agents at variance to proliferative PBs (42).

Cell phenotype described above came from studies conducted either in secondary lymphoid organs considered as the generation site, or blood considered as the circulation site, or BM considered as the long-term residency site. Several concerns exist arguing that the phenotype of ASC infiltrating autoimmune tissues may differ. Indeed, one may ask whether terminal differentiation as observed upon homing in the sterile environment of the bone marrow is also similarly occurring after infiltration in an inflamed autoimmune tissue. Autoimmune tissues may harbor ectopic germinal centers (EGCs) commonly defined by an aggregate of proliferating T and B cells intermixed with CD21^+^ follicular dendritic cells (FDCs) (43). In these cases, the tissue should contain different population of ASCs. All these considerations may highly modulate ASCs susceptibility to current biotherapies targeting B cells discussed above. Here, we will review the literature describing the phenotype and stage of differentiation of ASCs infiltrating autoimmune tissues. We will also review the chemokines engaged in ASC homing (44, 45). Among these chemokines, CXCL-12 has been frequently studied. One should note that this axis is not strictly specific to ASCs, since many leukocytes express the associated receptor, CXCR-4, and that dual pro- and anti-inflammatory functions has been associated to it (46, 47). We selected diseases based on the frequent tissue infiltration by ASCs and/or the description of pathogenic antibodies defined by their ability to transfer/exacerbate the disease in an animal model. We are also discussing kidney allotransplants rejected upon a chronic humoral immune response, since these tissues are displaying high *in situ* similarities with autoimmune tissues and allotransplanted tissues are also considered for B-cell depletion (48).

## 2 Systemic diseases

### 2.1 Systemic lupus erythematosus

SLE is an autoimmune disease characterized by multi-organ involvement and the production of autoantibodies that target nuclear self-antigens of two types. The first type includes antibodies to double-stranded (DS) DNA and nucleosomal components. The second type, named antibodies to extractable nuclear antigens (ENA), includes antibodies to RNA binding proteins. Anti-DS DNA antibodies play a crucial role in the inflammatory and fibrogenic mechanisms of lupus nephritis (LN), while immune complexes with anti-ENA antibodies stimulate interferon production by innate immune cells (49, 50). Pathogenicity of these antibodies was demonstrated in the human system during pregnancy with the neonatal lupus syndrome (51). All B-cell therapies discussed above were tested in SLE (52). However, only the anti-BAFF has been approved to treat patients. This may be explained by the strong correlation observed between serum levels of BAFF and disease severity. Notably, the anti-BAFF in use recognizes only the membrane-bound form of the molecule (53). The anti-CD20 has been disappointing in clinical trials, and a combination of the anti-BAFF and anti-CD20 is currently under evaluation (54). Most recently, telitaciept was approved for the treatment of active SLE in China (14). Epratuzumab failed in SLE, but a *post-hoc* analysis revealed efficacy in SLE patients with an associated Sjögren syndrome (SS) (55). Of note in SLE is an antibody against the type I IFN receptor recently approved (56). It is believed that such drug may affect ASC differentiation (57). Quite new in the autoimmunity field, a small cohort of refractory SLE patients has been successfully treated with CAR-T cells directed against CD19 (58).

In this systemic disease, tissue lesions with CD138^+^ ASCs may be observed in kidneys from LN (59). Beyond CD138, their precise phenotype has not been extensively studied. These cells express CXCR4, and CXCL-12 is upregulated in tubules and glomeruli of kidneys from LN patients (60, 61). A single cell RNA analysis recently confirmed the prevalence of the CXCL-12/CXCR4 axis for leukocyte infiltration in LN, and provided evidences that recruited leukocytes may also be a source of CXCL-12 in the inflamed tissue (62). EGC formation may be observed in these inflamed kidneys (63). In addition, expansion of effector B cells of extrafollicular origin may occur during flares (64). Interestingly, the predominance of GCs and extrafollicular B responses may vary among SLE patients (65). Regarding ASC survival factors, BAFF and APRIL have been detected in kidneys from LN (66). However, expression of their receptors has not yet been reported in kidney-infiltrating ASCs.

### 2.2 Anti-neutrophil cytoplasmic antibody-associated vasculitis

Anti-neutrophil cytoplasmic antibody (ANCA)-associated vasculitis (AAV) are represented by granulomatosis with polyangiitis (GPA) and microscopic polyangiitis (MPA). The antibodies are directed against cytoplasmic antigens, primarily proteinase 3 (PR3) and myeloperoxidase (MPO) from neutrophils. PR3-ANCA is associated with GPA (75%), whereas MPO-ANCA is more commonly associated with MPA (60%). Pathogenicity of ANCA was demonstrated in the human system with the transplacental transfer of MPO-ANCA and induction of renal pathologies (67). The anti-CD20 has been approved in conjunction with corticosteroids (68). Rituximab has also been licensed for remission induction in severe GPA/MPA and refractory/relapsing GPA/MPA (69). At variance, the anti-BAFF was disappointing in this disease (70). It is now tested in association with rituximab (NCT03967925).

Because of the systemic nature of this disease, ASCs have been mostly studied in the circulation. Matsumoto et al. have shown that AAV patients display higher proportions of circulating PBs and PCs as compared to healthy controls, and von Borstel et al. demonstrated that an increased frequency of these cells defined by expression of CD27 and CD38 in GPA patients during remission is related to a higher relapse risk (71, 72). The kidney may be involved in AAV with tubulointerstitial lesions containing inflammatory cells, and a case report showed structures similar to EGCs with an aggregates of T/B cells surrounded by CD138^+^ PCs (73, 74). To our knowledge, nothing was reported for *in situ* chemokine production, infiltrating ASCs and BAFF/APRIL expression in kidney lesions from AAV.

### 2.3 IgG4-related diseases

IgG4-related diseases (IgG4-RDs) are antibody-mediated disorders not considered as strictly of autoimmune origin but rather as a wide spectrum of fibro-inflammatory conditions affecting multiple organs and associated with infiltration of ASCs specifically producing IgG4. Nevertheless, test of patients’serum reactivity defined several autoantigens including prohibitin, annexin A11, laminin 511-E8 and galectin-3 (75). IgG4-RD involves several unrelated organs such as the pancreas, liver, kidney, lung and skin. Despite the fact that the IgG4 isotype is not able to bind complement proteins and activate Fc receptors present on immune cells, pooled IgG4 from patients on their own are nevertheless able to induce pancreatic and salivary gland injuries when injected subcutaneously in neonate mice (76). It is believed that IgG4 pathogenic autoantibodies exert their effect by an antagonism activity of the targeted autoantigen(s). As a relevant example, recombinant antibodies produced from circulating clonally related PBs defined IL-1 receptor antagonist (IL-1 RA) as a new autoantigen, and these antibodies showed an antagonistic activity against IL-1 RA (77). It should be noted that an heterogeneity exist at the level of the isotype of pathogenic autoantibodies produced in this disease, since serum IgG1 from patients also transferred the diseases in neonate mice (76). Rituximab, belimumab associated to prednisone and obexelimab, a second form of anti-CD19, are in trials (NCT01584388, NCT04660565, NCT05662241).

Ducts and acini produce CXCL-12 in IgG4-RDs affecting the pancreas (78). The receptor CXCR4 has also been detected in pancreatic lesions, but the type of cells expressing this receptor were not studied. As for AAV, elevation in total circulating PBs defined in this case by a CD19^low^CD20^-^CD38^high^CD138^-^ phenotype is a valuable biomarker in IgG4-RDs (79). Among these cells, more than half are IgG4^+^. EGCs are frequent in IgG4-RDs (80). The precise phenotype of infiltrating IgG4^+^ ASCs remains to be determined, since only CD138 expression was assessed in kidney and skin lesions (81, 82). While there are no data on BAFF expression in IgG4-RDs, APRIL is produced by CD163^+^ M2 macrophages (83). There are no data on BAFF-R/TACI/BCMA expression by infiltrating ASCs.

## 3 Organ-specific diseases

### 3.1 Sjögren’s syndrome

Primary Sjögren’s syndrome (pSS) is an autoimmune disease affecting among others salivary and lacrimal glands with 2/3 of the patients harboring the so-called anti-Ro/SSA and anti-La/SSB antibodies recognizing ribonucleoproteins (84). Despite their intracellular reactivities, these autoantibodies may be pathogenic, since maternal antibodies transferred *via* the placenta induce cutaneous lesions in neonates (85) Regarding B-cell therapies, rituximab gave contradictory results but some positive clinical efficacies could be highlighted (86). The anti-BAFF and anti-BAFF-R gave promising results in phase II (87, 88). The anti-BAFF is also currently tested associated to the anti-CD20 (NCT02631538). A trial with telitacicept was recently announced (NCT05673993). A trial with CAR T cells co-targeting CD19 and BCMA has been announced in refractory SS patients (NCT05085431).

**Figure 1:**
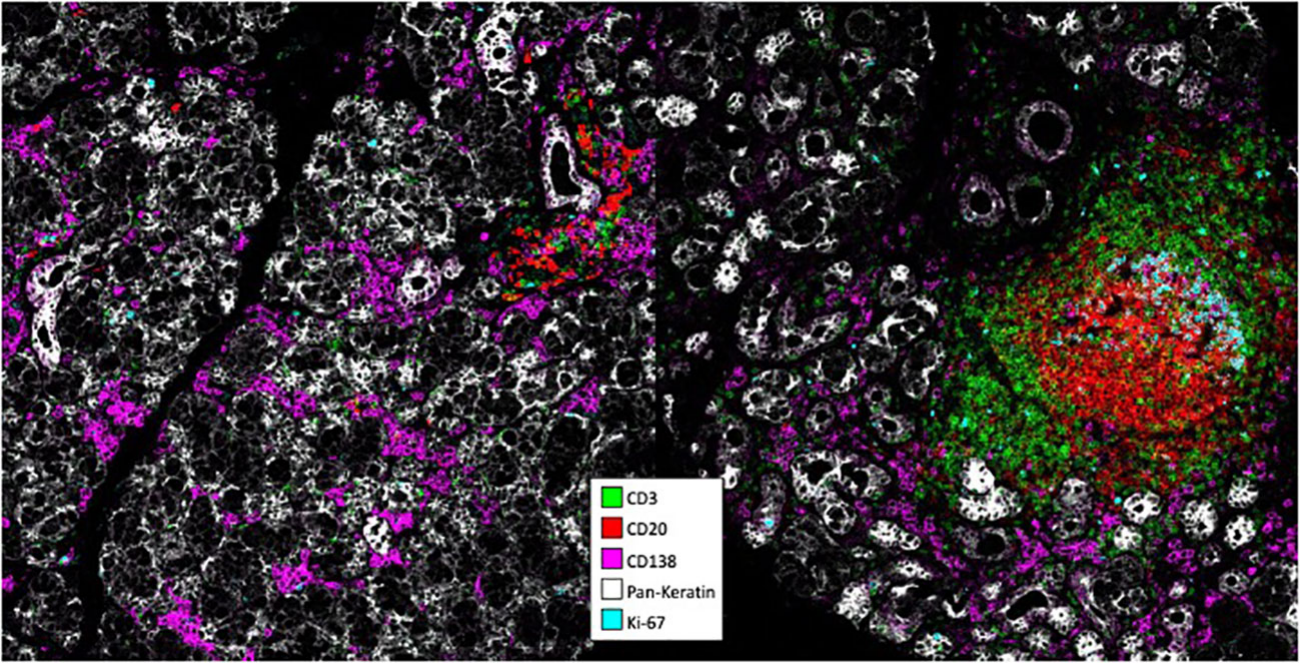
Analysis of pSS lesions with imaging mass cytometry. Formalin-ixed paraffin-embedded minor salivary gland biopsies corresponding to one pSS patient with mild infitration and one patient with an ectopic germinal center were stained with antibodies conjugated to metal tags using the MaxPar® labeling kit. Stainings were visualized on a Hyperion. Imaging system coupled to a Helios mass cytometer.

pSS has a frequent association with EGCs averaging 30-40% (43). These EGCs have been associated to SS threatening complications with the development of B-cell lymphomas (89). ASCs are well present even in lesions with a low focus score devoid of EGCs (90). Regarding homing, ASCs are found in the vicinity of CXCL-12 expressing cells including epithelial cells, infiltrating leukocytes and adipocytes (91). Notably, CXCL-12 upregulation was also seen in cases with EGCs, implying that it may not only act as a recruitment but also as a retention factor. Another study reported an upregulation of CXCL-9/-10 in pSS compared to healthy salivary glands (92). In EGC^+^ cases, most ASCs identified by their CD138 expression did not proliferate based on Ki67 reactivity. This observation is of particular importance, and we recently confirmed this finding by imaging mass cytometry with most if not all CD138^+^ cells outside EGCs negative for Ki67 (**Figure 1**, data not published). Indeed, it shows that ASCs produced locally differentiate further than the PB stage in the autoimmune tissue. Notably, it has been possible to identify in this disease autoreactive ASCs in lesions by using tagged recombinant autoantigens as detection reagents. By this approach, ASCs binding the Ro52 and Ro60 autoantigens were described among CD20^-^CD19^+^ cells (93–95). Regarding survival factors, pSS is one exception among autoimmune tissues characterized by the absence of APRIL expression, whose expression is usually associated with inflammation (96). At variance, BAFF is well expressed, but the expression of BAFF-R on ASCs of pSS has not yet been assessed (97).

### 3.2 Rheumatoid arthritis

RA is an autoimmune disease showing inflammation in the synovial tissue and affecting joints. RA is associated with autoantibodies against citrunillated protein antigens (ACPA). Anti-ACPA are present in more than 80% and 50% in advanced and early RA, respectively. A common mouse model for RA is the transfer of an antibody against the glucose-6-phosphate isomerase into mice (98). As discussed earlier, RA was the first autoimmune disease with an approved B-cell therapy in the form of an anti-CD20. Three phase II trials were performed with atacicept with only one showing a positive clinical response (99). Adding atacicept to rituximab did not ameliorate efficacy (100) A phase II study showed promising results with the anti-BAFF (101). The anti-BAFF-R is in trial (NCT03574545).

As for pSS, EGCs are frequent in RA averaging 40% (43). EGCs are necessarily associated with increased synovium inflammation that may extend systemically, but not necessarily associated to the presence of anti-ACPA (102). ASCs may also be present even in the absence of these EGCs in the early phase of the disease (103). The chemokines CXCL-9/-10 together with expression of their receptor CXCR3 on ASCs have been detected in the synovial tissue of patients (104, 105). CXCL-12 is also upregulated, and CXCR-4 has been reported in ASCS from synovial tissues at the mRNA level (106). Notably, Scheel et al. reported that B cells may differentiate into ASCs within synovial tissues devoid of EGCs (107). In cases positive for EGCs, ASCs present outside EGCs have a non-proliferating phenotype being CD20^-^ and expressing high level of CD38 and CD138 (108, 109). This confirms the observation made in pSS indicating that ASCs may differentiate to the PC stage in an autoimmune tissue. However, a recent single cell RNAseq analysis combined to mass cytometry classifies the majority of ASCs present in synovial tissues as PBs (110). Autoreactivity of ASCs in RA was also probed with tagged-ACPA, and showed that a subset of CD20^-^CD138^+^ ASCs residing outside EGCs were producing autoantibodies (111). Autoreactivity against ACPA was further confirmed with the recombinant antibody technology (112). These ASCs in RA lesions were probed t be non-susceptible to rituximab treatment (113). BAFF and APRIL produced by synovial fibroblasts as well as myeloid cells in the case of APRIL are present in inflamed synovial tissues (114–117). Data regarding BAFF-R, TACI and BCMA expression on ASCs at the protein level are missing. However, BCMA and TACI has been described in ASCs from synovial tissues at the mRNA level (112).

### 3.3 Liver autoimmune diseases

The liver may suffer from an autoimmune attack in mainly two different situations, the autoimmune hepatitis (AIH) and the primary biliary cholangitis (PBC). Two types of AIH have been described according to autoantibody reactivity in patient serum. Type 1 AIH is defined by anti-smooth muscle antibodies directed against actin filaments and associated or not with anti-nuclear antibodies. Type 2 AIH is associated to autoantibodies more specific to the tissue with the so-called liver-kidney microsomal 1 directed against cytochrome P450-2D6, and/or liver-cytosol 1 targeting forminotransferase-cyclodeaminase antibodies. Autoantigens in PBC originate from the mitochondria. AIH-like symptoms were reproduced in mice by passive transfer of an antibody reactive against the hepatocyte surface of IgM isotype and derived from a type 1 AIH patient (118). To our knowledge, induction of PBC by transfer of anti-mitochondrial antibodies from patients in an animal model has not been reported. Quite surprisingly, there is only the anti-BAFF-R in trial for AIH. This is likely explained by the fact that steroid and/or anti-mitotic agents are highly effective in AIH with a limited number of patients estimated around 10% failing. Rituximab and to a lesser extent belimumab were tested with some promising successes in steroid non-responding patients from non-randomized trials (119–122). Notably, rituximab was reported effective in cases of refractory AIH/PBC overlap (123). However, no clinical trials have been announced yet. Rituximab failed in phase II for PBC despite reduction in anti-mitochondrial antibodies (124). To the best of our knowledge, EGCs have not been defined in AIH. Despite this, ASC infiltration is common, ranging to about 2/3 of cases (125). Since seropositivity for autoantibodies is now considered to be superior to 90% in AIH if one considers all autoantibodies described in this disease and not only the type-specific ones (126), seropositive AIH is composed of a least two subtypes, one with and one without ASC infiltration. There are no data regarding ASC specific chemokines in AIH lesions. In addition to the absence of EGCs, this is raising questions regarding ASC homing in AIH livers. CD138^+^ PCs are observed in AIH lesions (127), but their precise phenotype warrants further investigations. At variance, ASC chemokines including CXCL-9/-10/-12 have been reported in PBC livers (128, 129). As a likely consequence, ASCs also infiltrate PBC livers. ASC infiltration is one common histological hallmark between AIH and PBC. One difference is the more prominent fraction of ASCs secreting switched IgG over IgM antibodies in AIH observed by several independent groups (130–132). Despite the fact that no studies are reporting T/B aggregates with the presence of CD21^+^ FDCs, EGCs are likely in PBC according to the study reporting the presence of PD-1^+^ ICOS^+^ T follicular helper (TFH) aggregated with CD20^+^ B cells (133). In PBC, a significant fraction of CD38^high^CD138^+^ ASCs expresses CD19 (134). These ASCs secrete antibodies against the PDC-E2 mitochondrial antigen. There is currently no data on the production of BAFF and APRIL in autoimmune livers.

### 3.4 Autoimmune thyroiditis

The thyroid may be subjected to a humoral autoimmune reaction with the Hashimoto thyroiditis (HT) and Graves disease (GD). HT and GD harbor anti-thyroperoxydase/thyroglobulin and anti-thyroid stimulating receptor, respectively. GD may be further associated to orbitopathy. Pathogenicity of these autoantibodies has been clearly established in the human system with spontaneous abortion problems (135, 136). Hypothyroidism in HT is well managed by hormone substitution therapies. Clinical management of GD is more complex, especially in the case of associated orbitopathy. Rituximab was first tested in GD not associated to orbitopathy. A decrease in pathogenic antibodies despite clinical improvement in some patients was not always correlated (137–139). This suggests another role for B cells than the production of autoantibodies in the pathogenesis of GD. Two clinical trials were achieved with rituximab in GD-associated orbitopathy giving controversial effects (140, 141). Rituximab may be effective when administered early in disease active patients, but additional data are needed before any conclusive statement. Two other phase II trials are ongoing.

ASC infiltration and EGC genesis is a very common feature of HT, and they are a source of autoantibodies (142). EGC genesis may also occur but to a lesser extent in GD (143). Regarding ASC chemokines, CXCL-9/-10 and CXCL-9/-10/-11 produced by thyrocytes have been reported in HT and GD, respectively (144–146). CD138 staining was not extensively studied in autoimmune thyroiditis, most likely because it is also expressed at the surface of thyrocytes. One study reported CD138^+^ PCs in GD (147). 15% of GD associated orbitopathy cases show a selective ASC infiltration in the orbital tissue (148). Notably, upregulation of CXCL-12 in the ocular manifestation of GD has been reported (149). BAFF is produced in the EGCs from HT (150). There is no data regarding APRIL.

### 3.5 Multiple sclerosis

The presence of oligoclonal immunoglobulin bands in the cerebrospinal fluid (CSF) of patients and the success obtained by B-cell therapies cited in the introduction led to an increased interest in the targeting of humoral immunity in multiple sclerosis (MS). Since the ocrelizumab approval, trial with the anti-CD19 gave trendy results (151). Atacicept failed in MS because of an unexpected disease exacerbation (152). Animals models provided several explanations (153–155). Nevertheless, atacicept failure might not have been directly due to BAFF/APRIL antagonism, since a new trial with telitacicept is ongoing in MS (14). BAFF-only treatment with tabalumab gave no significant results (156). As of today, the disease has never been transferred in an animal with the systemic injection of antibodies directed against myelin-associated products (157). However, systemic injection of these autoantibodies decreased the number of encephalitogenic T cells required to induce demyelinating lesions in the mouse CNS, indicating that the humoral immunity may not induce the disease but participates in its propagation. In patients, one subtype of MS is associated with antibody/complement-mediated demyelination (158). All together, these confirm the potential value to target ASCs in MS.

Non-proliferative terminally differentiated CD138^+^ PCs have been reported in the CNS parenchyma from MS patients (159). However, most of the reports regarding local ASCs in MS concerns the cerebrospinal fluid (CSF). CXCL-12 was reported upregulated in the parenchyma and CSF of MS patients (160). CSF ASCs expressed CD138, but were nevertheless classified as PBs due to their coexpression of CD19 and HLA-DR (161). While CD19 expression might now be controversial as discussed earlier to distinguish ASC differentiation stages, expression of HLA-DR has been excluded from the PC stage, at least in bone marrow (162). These PBs were detected throughout the disease course in patients, and their number correlated to the level of intrathecal immunoglobulins. This indicates that short-lived PBs are the main ASCs in CSF from MS patients. Arguing in favor of the later, a continuous replenishment of these cells was evidenced by B-cell differentiation into ASCs within the CSF (163, 164). Furthermore, EGCs containing an aggregate of T and B cells, a network of CD21^+^ FDCs, and surrounded by CD138^+^ ASCs have also been detected in meninges of MS patients presenting the secondary progressive form of the disease (165, 166). Upregulation of BAFF and APRIL in the CSF of MS patients is controversial (167, 168). While there is no direct data about the expression of BCMA/TACI on the surface of *in situ* ASCs, the elevated presence in the CSF of protease-cleaved soluble BCMA and TACI, a natural process regulating their surface expression, indicates that they may be expressed on CSF ASCs (169, 170). We and others detected BAFF and APRIL expression produced by astrocytes and myeloid cells, respectively, in MS lesions (155, 171). There, APRIL fulfills an immune function outside humoral immunity by triggering an IL-10-based immunosuppressive response from reactive astrocytes. It should be noted that two independent studies in the gold standard mouse model of MS, the experimental autoimmune encephalitis, revealed ASCs harboring an unexpected immunosuppressive function own to their expression of immunosuppressive cytokines such as IL-10. One group provided strong evidences for migration into the CNS during the disease course of gut-derived IL-10-producing IgA^+^ ASCs (154). The other group described regulatory ASCs producing IL-10 and IL-35, another cytokine with immunosuppressive function, in secondary lymphoid organs (172). Notably, ASCs producing IL-10 have been detected in CNS lesions from MS patients (173, 174). The phenotype of these cells has not yet been assessed in detail.

### 3.6 Neuromyelitis optica spectrum disorders

NMO is nowadays considered part of a spectrum disorders (NMOSD) of autoimmune origin affecting the central nervous system, targeting astrocytes and conducting to acute myelitis, optic neuritis and encephalitis. One hallmark of NMO is the presence of IgG autoantibodies to aquaporin 4, a receptor present at the surface of foot processes of astrocytes. Disease transfer by administration of IgG anti-aquaporin 4 from patients was demonstrated by several groups (175). As introduced above, inebilizumab has been approved in NMOSD, and rituximab is in trial (176).

CSF from NMO patients contain elevated levels of CXCL-10 (177, 178). IgG^+^ ASCs with a PB phenotype according to their expression of CD19 and HLA-DR are present in NMO CSF (179). As in MS, ASC differentiation in patients’ CSF has been proposed. BAFF and APRIL are upregulated in NMO CSFs (167, 180). EGCs in the orbital mass have been reported in two patients (181). These EGCs were defined by an accumulation of CD20^+^ B lymphocytes, BCL6 expression and surrounding ASCs of IgG and to a lesser extent IgG4 isotypes. As in MS, APRIL targets reactive astrocytes in NMO lesions (182).

### 3.7 Kidney allotransplants

The main challenge in solid organ allotransplantation including the kidney remains the late humoral rejection. These antibody-mediated rejections (ABMR) are the consequence of the generation of donor-specific antibodies (DSA) against mismatched human leukocyte antigens. Near 20% of the kidney recipients may develop *de novo* DSA by 5 years post transplantation, which leads to a high risk of graft dysfunction and a low graft survival as compared to transplantation recipient without DSA. Pathogenicity of DSA has been well established when allotransplantations were unsuccessfully performed in recipients with preformed anti-DSA. The later resulted in a mandatory donor sensitization with plasma exchange prior to graft. Correlative studies indicated that IgG3 and IgG4 anti-DSA are the two main isotypes involved in ABMR (183, 184). Rituximab-based regimen were shown to improve graft survival in 4/7 acute ABMR but only 1/7 chronic ABMR (185). The subsequent clinical trial failed for acute ABMR (186). Belimumab is promising with the reduction of *de novo* DSA antibody formation in a phase II trial (187). Bortezomib has been tested in late/chronic ABMR with some promising results, but the following randomized clinical trial was disappointing (188). Hence, PC targeting in late ABMR is a real concern (189). Recently, a case report with a kidney allograft patient further diagnosed with smoldering myeloma, an early form of MM, reported promising results upon treatment with daratumumab according to levels of anti-DSA and graft survival (190).

Regarding chemokines, CXCR3 with an upregulation of CXCL-11 is thought to be involved in many allograft rejections including kidney (191, 192). CXCL-12 is also elevated in chronic rejection (193). In acute rejection, ASCs are thought to be scarce accounting usually for less than 5% of the cellular infiltrate (194). Nevertheless, presence of these cells has been associated with a poor allograft function and survival (195). One study reported few cases (3%) with numerous CD138^+^ PCs accounting for up to 30% of the infiltrate (196). ASC-rich infiltrates tend to occur late in the rejection process. ECGs defined by an aggregate of TFH and B cells have been described in acute rejection (197). Longitudinal studies are doable in allograft patients. Proliferative lymphocyte aggregates without FDCs were reported already present in almost 50% of biopsies performed one month after transplant in clinically stable graft (198). EGCs reached 19% at 12 months, and were associated with progressive graft dysfunction. EGCs in kidney allografts appear to be resistant to rituximab treatment (199). In chronic rejection, a local production of DSA against HLA class I and II molecules was evidenced (200, 201). Both BAFF and APRIL are present *in situ* in ABMR (202).

## 4 Concluding remarks

It is commonly accepted that human clinical diseases are not single entities but composed of several subtypes. This is well-known also in autoimmune diseases. **Table 1A** highlights that autoimmune diseases highly differ in their susceptibility to current drugs targeting the humoral immunity. If one assumes that the biodistribution of all these antibody-based biotherapies is similar for a given tissue, **Table 1** shows that diseases are biotherapy specific. When ASCs infiltrating the autoimmune tissues are considered, heterogeneity exists at several levels. The first one is the tissue of origin. It could the draining secondary lymphoid organs associated to the production of chemokines within the tissue. **Table 1B** shows that chemokines may be different according to the tissue considered. Alternatively, it could be inside the pathologic tissue with either clonal expansion of differentiated cells or the differentiation of precursor cells within EGCs. Regarding the latter, their presence appears to be predominant in autoimmune tissues, since only one disease, AIH, has not yet been described to contain EGCs as indicated in **Table 1B**. It is difficult to state if there is a specific timing of occurrence for EGCs during an autoimmune disease course without the access to longitudinal biopsies from the considered tissues. However, study in kidney transplantation revealed that EGC formation may be frequent in a tissue targeted by an immune reaction.

Table 1ABiotherapies targeting the humoral immunity.

**Table:** 

Diseases	Biotherapy approved	Biotherapy failed	Biotherapy tested
LN	Anti-BAFF (6), Soluble TACI (14)	Anti-CD20 (54), Anti-CD22 (55)	Anti-BAFF+Anti-CD20 (NCT03747159/phase Ill)
AAV	Anti-CD20 (68, 69)	Anti-BAFF (70)	Anti-BAFF+anti-CD20 (NCT03967925)
IgG4-RD	None	None	Anti-CDI9 (NCT056622421/phase II)
			Anti-CD20 (NCT015S4388/phase 1-11)
			Anti-BAFF (NCT0116066/phase II)
pSS	None	Anti-CD20 (86)	Anti-BAFF (NCTOll606661phase IIb)
			Anti-BAFF-R (NCT02962S95/phase IIb)
			Anti-CD20+anti-BAFF (NCT0263153S/phase II) soluble TACI
			(NCT05673993/pbase Ill)
RA	Anti-CD20 (I)	None	Anti-BAFF (NCT00071S12Jphase II)
			anti-BAFF-R (NCT03574545/phase I)
			soluble TACI (NCT00595413/pbase II, NCT00664521/phase II,
			NCT00430495/phase II, NCT03016013/phase III)
AIH	None	None	anti-BAFF-R (NCT03217422/phase II)
PBC	None	Anti-CD20 (124)	None
HT	None	None	None
GD	None	None	Anti-CD20 (NCT0015011l/phase II, NCT00424151/phase II,
			NCT00595335/phase II, NCT02378298/phase IV)
MS	Anti-CD20 (5)	Anti-BAFF (156), Soluble TACI (152)	Soluble TACI (NCT04625153/pbase II)
NMO	Anti-CD19 (10)	None	Anti-CD20 (NCT04256252/phase IV, NCT00501748/phase I)
ABMR	None	Anti-CD20 (186)	Anti-BAFF (NCT01536379/phase II)

Biotherapies approved, failed and currently tested are listed. References are given for approved and failed biotherapies. Registration numbers from ClinicalTrials.gov for ongoing clinical trials are also given.

**Table 1B:** Heterogeneity in ASC infiltrating auto/alloimmune tissues.

Diseases	Chemokines	Stage	Auto/alloreactivity	Isotype Ig	EGCs
**LN**	CXCL-12	PC/CD138+	?	?	yes
**AAV**	?	PC/CD138+	?	IgG	yes
**IgG4-RD**	CXCL-12	PC/CD138+	?	IgG4, IgG1	yes
**pSS**	CXCL-9,-10,-12	PC/CD138+ Ki67-	yes	IgG	yes
**RA**	CXCL-9,-10,-12	PC/CD138+ Ki67-	yes	undefined	yes
		PB/CD38	?		
**AIH**	?	PC/CD138+	?	IgM	no
**PBC**	CXCL-9,-10	PC/CD138+	yes	?	yes
		PC/CD138+CD19+	yes		
**HT**	CXCL-9,-10,-12	?			Yes
**GD**	CXCL-9,-10,-11	PC/CD138+	yes	IgG4, IgG1	Yes
**MS**	CXCL-12	PB/CD138+CD19+HLADR+	?	IgG	yes
**NMO**	CXCL-10,-13	PB/CD138+CD19+HLADR+	no	IgG	yes
**ABMR**	CXCL-11,-12	PC/CD138+	yes	IgG3/IgG4	yes

Tissue chemokines acting on plasma cells, putative stages of differentiation according to the indicated markers, specificity (auto/allo), isotype of the pathogenic antibodies secreted and presence of ectopic germinal center within the inflamed tissue are listed.

ASCs infiltrating autoimmune tissues have been convincingly shown to produce antibodies targeting autoantigens in several autoimmune diseases (**Table 1B**). This highlights their contribution, at least in part, to the pathogenicity, and warrants their targeting to test treatment improvement. However, phenotype of these cells is another level of heterogeneity rendering their targeting complex. Indeed, isotypes of the pathogenic antibodies first vary (**Table 1B**). More importantly, their stage of differentiation may also vary with the report of PBs and PCs (**Table 1B**). The latter shows that ASCs have the ability to complete their full differentiation process into PCs in some tissues. In total, an extensive phenotype characterization of ASC infiltrating autoimmune tissues now possible with high throughput technologies is warranted before any efficient ASC-targeting treatment could be designed. This approach is ongoing, and should bring key molecular targets in the near future.

## Author contributions

BH designed the study and wrote the manuscript. DG, ABe, ABa, LB, MK, CD-P, GC, JN, J-OP, NS wrote the manuscript. All authors contributed to the article and approved the submitted version.
